# A Review of Machine Learning and Algorithmic Methods for Protein Phosphorylation Site Prediction

**DOI:** 10.1016/j.gpb.2023.03.007

**Published:** 2023-10-19

**Authors:** Farzaneh Esmaili, Mahdi Pourmirzaei, Shahin Ramazi, Seyedehsamaneh Shojaeilangari, Elham Yavari

**Affiliations:** 1 Department of Information Technology, Tarbiat Modares University, Tehran 14115-111, Iran; 2 Department of Biophysics, Faculty of Biological Sciences, Tarbiat Modares University, Tehran 14115-111, Iran; 3 Biomedical Engineering Group, Department of Electrical Engineering and Information Technology, Iranian Research Organization for Science and Technology (IROST), Tehran 33535-111, Iran

**Keywords:** Phosphorylation, Machine learning, Deep learning, Post-translational modification, Database

## Abstract

**Post-translational modifications** (PTMs) have key roles in extending the functional diversity of proteins and, as a result, regulating diverse cellular processes in prokaryotic and eukaryotic organisms. **Phosphorylation** modification is a vital PTM that occurs in most proteins and plays a significant role in many biological processes. Disorders in the phosphorylation process lead to multiple diseases, including neurological disorders and cancers. The purpose of this review is to organize this body of knowledge associated with phosphorylation site (p-site) prediction to facilitate future research in this field. At first, we comprehensively review all related **databases** and introduce all steps regarding dataset creation, data preprocessing, and method evaluation in p-site prediction. Next, we investigate p-site prediction methods, which are divided into two computational groups: algorithmic and **machine learning** (ML). Additionally, it is shown that there are basically two main approaches for p-site prediction by ML: conventional and end-to-end **deep learning** methods, both of which are given an overview. Moreover, this review introduces the most important feature extraction techniques, which have mostly been used in p-site prediction. Finally, we create three test sets from new proteins related to the released version of the database of protein post-translational modifications (dbPTM) in 2022 based on general and human species. Evaluating online p-site prediction tools on newly added proteins introduced in the dbPTM 2022 release, distinct from those in the dbPTM 2019 release, reveals their limitations. In other words, the actual performance of these online p-site prediction tools on unseen proteins is notably lower than the results reported in their respective research papers.

## Introduction

Post-translational modifications (PTMs) are biochemical reactions occurring on a protein after its translation [1], [2], which change the regulated physicochemical properties, maturity, and activity of most proteins [3], [4]. It includes cutting, folding, ligand-binding, adding a modifying group to one or more amino acids, and finally changing the chemical nature of amino acids [5], [6]. In recent years, an increasing volume of PTM data has become available because of improvements in mass spectrometry (MS) based on high-throughput proteomics [7]. There are more than 600 types of PTMs [8] that affect many aspects of cellular functionalities, such as metabolism, signal transduction, activity, stability, and localization of various proteins [9], [10]. Recent studies have shown that each modification leads to a multitude of effects on the structure and, therefore, the function of the proteins [11]. PTMs include phosphorylation, glycosylation, ubiquitination, sumoylation, acetylation, succinylation, and nitrosylation, as well as numerous others involved in most cellular activities [9], [12], [13], [14], [15], [16]. Moreover, PTMs play key roles in a variety of biological regulatory pathways, including metabolic pathways,  DNA damage response, transcriptional regulation, signaling pathways, protein–protein interactions (PPIs), apoptosis, cell death, insulin signaling, immune response, and aging [17], [18]. The dysregulation of PTMs is linked to diseases such as cancer, diabetes, cardiovascular disease, and neurological disorders [19], [20], [21], [22], [23], [24].

Phosphorylation is one of the most important reversible PTMs. Phoebus Levene discovered phosphorylation in 1906 in the protein vitellin (phosvitin) [25]. In phosphorylation, a −2 phosphate group is covalently added to serine (S), threonine (T), tyrosine (Y), and histidine (H) residues and then removed by protein phosphatases. It is known that protein phosphorylation regulates the activity of various enzymes and receptors, including signal pathways [26], and can greatly impact the folding, function, stability, and subcellular localization of the protein [25], [27], [28]. In eukaryotes, this modification plays a vital role in signal transduction and other biological functions, including protein synthesis, cell division, signal transduction, DNA repair, environmental stress response, transcriptional regulation, apoptosis, cellular motility, immune response, metabolism, cell growth, development, cellular differentiation, and aging [29], [30].

In eukaryotes, the phosphorylation process is catalyzed via protein kinases (PKs) differentially and specifically in which each PK only modifies a subset of substrates to ensure signaling fidelity [31]. The phosphorylation process typically occurs on T, S, and Y residues. However, in prokaryotes, plants, and fungi, this modification also occurs on H and aspartate (D) residues in protein sequences, which play important roles in two-component signaling systems [32], [33]. Indeed, phosphohistidine (PhosH) is involved in many biological processes, such as central metabolism and signaling in eukaryotes and bacteria, making it important to develop and potentially improve computational tools for accurately predicting histidine phosphorylation sites (p-sites) [32], [33].

Phosphorylation is present in more than one-third of human proteins, and this modification is regulated by approximately 568 human PKs and 156 protein phosphatases [29]. In this sense, phosphorylation is one of the widest spread and most extensively studied protein PTMs, which has a significant role in the control of biological processes like proliferation, differentiation, and apoptosis [29], [34]. Site mutations or dysregulation of kinase activity, their hyperactivity, malfunction, or overexpression, and also the hyperphosphorylation of human proteins are associated with certain disease states such as cancer, Alzheimer’s disease (AD), Parkinson’s disease (PD), frontotemporal dementia (FTD), and various pathways involving the immune system [27], [28], [29], [35]. Therefore, identifying kinase-specific p-sites is essential for understanding the regulatory mechanisms of phosphorylation.

**Figure 1:**
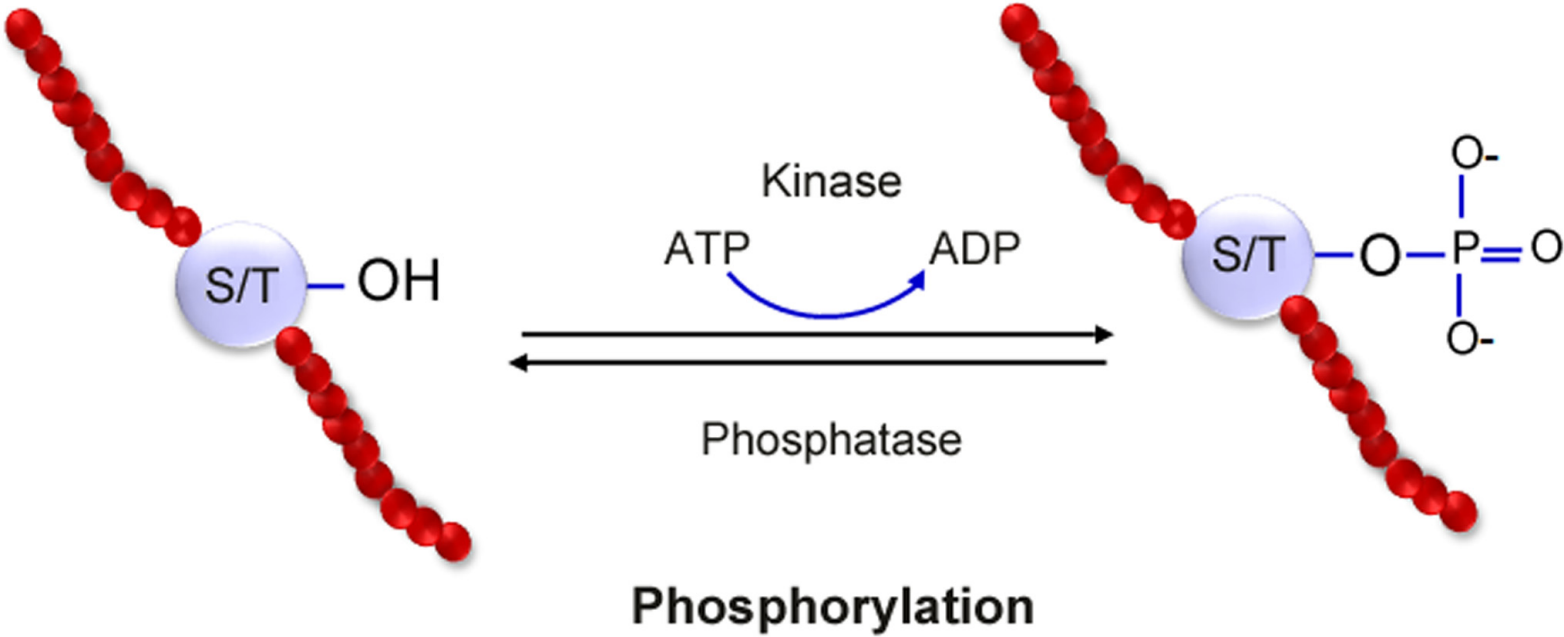
**Schematic of protein phosphorylationprocess** The red balls represent amino acids. ATP, adenosine triphosphate; ADP, adenosine diphosphate.

Multiple experimental methods are used for the detection assays of protein phosphorylation, like liquid chromatography-tandem mass spectrometry (LC-MS/MS), radioactive chemical labeling, and immunological detection [such as proximity ligation assay (PLA), chromatin immunoprecipitation, and Western blotting], although the combination of the LC-MS/MS method with the immunoprecipitation (IP) strategy is suitable for the detection of p-sites in proteins [36], [37]. Cellular processes are regulated by phosphorylation, which is highly conserved and significantly affects protein stability. Figure 1 shows the schematic of protein phosphorylation process.

The number of known p-sites has grown since 2003, and it rose from 2000 to more than 500,000 in experimental databases. The p-sites are involved in the regulation of at least 30% of human proteomes [38], [39].

Indeed, experimental approaches are generally difficult, slow, and costly, and need specialized equipment and knowledge. Over the last two decades, PTM research has made remarkable progress due to technological advancements and the emergence of new computational methods.

A previous study [36] has reviewed PTM tools, resources, and related databases and also investigated the challenges of algorithmic methods. The authors divided 10 types of PTMs into small chemical groups, lipids, and small proteins, and they investigated databases and algorithmic approaches for different PTM sites [36]. Shi et al. [40] reviewed 19 available tools for phosphorylation networks. They reported different analyses for their functionality, data sources, performance, network visualization, and implementation. Rashid et al. [41] reviewed specified machine learning (ML) methods, main feature selection methods, databases, and current online tools for microbial p-sites. They only investigated microbial p-sites and did not mention other p-sites in organisms nonetheless. Also, their work was limited to classical ML methods.

In this study, unlike other previous studies, we investigated all features, databases, and methods concerning p-site prediction. First, valid PTM databases that contain phosphorylation experimental data were introduced. Then, the two most important phosphorylation databases were reviewed, in which the numbers of organisms and p-sites were covered in detail. Furthermore, two main data preparation steps for p-site datasets, including data collection and data preprocessing, were reviewed. In other words, this study investigated methods for data collection and also introduced the most important and functional approaches for data preprocessing. Additionally, all evaluation metrics that have been used for p-site prediction were introduced. Then, the most common and important feature extraction methods were described based on the physicochemical, sequence, evolutionary, and structural properties of amino acids. It was found that there are generally two ML-based approaches for p-site prediction, which are divided into conventional ML and end-to-end deep learning (DL) methods. In the present study, the methods of both approaches were reviewed, and the available online tools for p-site prediction were briefly introduced.

Finally, we created three test sets from new proteins related to the released version of the database of protein post-translational modifications (dbPTM) in 2022, and then evaluated and compared the available online tools together in different metrics on the three specific test sets.

## Databases

Developing a prediction model requires a dataset of experimentally-validated p-sites. Therefore, the availability of general and specific databases for p-sites is the first step toward this end [36]. Databases are constantly evolving due to the advent of technology and may be updated by providing accurate details. These databases contain information about different organisms, such as viruses, animals, and plants, that has been collected manually and experimentally. For instance, all the information in the Human Protein Reference Database (HPRD) has been collected manually, and it contains more than 95,000 p-sites extracted from ∼ 13,000 proteins [42].

Considering different types of PTMs, databases are arranged into specific and general terms, in which general PTM databases cover a wide domain of data for different types of PTM, but specific databases are constructed based on special types of PTMs like phosphorylation.

Databases such as dbPTM [43], SysPTM [3], Swiss-Prot [44], and HPRD [42] are general databases that cover different types of PTMs, and phosphorylation is one of them. On the other hand, the eukaryotic phosphorylation site database (EPSD) [45], LymPHOS2 [46], Phospho3D [47], Phospho.ELM [48], and Regulatory Network in Protein Phosphorylation (RegPhos) [49] are specifically gathered for p-sites.

In the following, two important databases, dbPTM and EPSD, which are known as general and specific databases for p-site data, are going to be introduced. Furthermore, Table 1 summarizes both general and specific databases according to their statistical information for p-sites.

**Table 1:** Summary of phosphorylation databases

**Type**	**Acronym**	**General statistic**		**Type of data and database**	**URL**
		**No.of covered organisms**	**No.of p-sites and phosphoproteins**		
General database	dbPTM [43]	More than 1000 organisms	p-site: ∼ 1,770,000 P: ∼ 557,700	Experimentally-validated and predicted; secondary	https://awi.cuhk.edu.cn/dbPTM/
	PhosphoSitePlus [50]	26 organisms	p-site: ∼ 240,000 P: ∼ 20,200	Experimentally-validated; primary	https://www.phosphosite.org
	PTMcode v2 [51]	19 organisms	p-site: ∼ 316,500 P: ∼ 45,300	Experimentally-validated; secondary	http://ptmcode.embl.de
	qPTM [52]	Human	p-site: ∼ 199,000 P: ∼ 18,402	Experimentally-validated; secondary	http://qptm.omicsbio.info/
	HPRD [42]	Human	p-site: ∼ 1100 P: ∼ 30,000	Experimentally-validated; primary	http://www.hprd.org
	PHOSIDA [53]	9 organisms	p-site: ∼ 70,000 P: ∼ 28,700	Experimentally-validated; secondary	–
	PTM-SD [1]	7 model organisms	p-site: ∼ 1600 P: ∼ 842	Experimentally-validated; secondary	http://www.dsimb.inserm.fr/dsimb_tools/PTM-SD
	SysPTM 2.0 [3]	6 organisms	p-site: ∼ 353,000 P: ∼ 53,200	Experimentally-validated; secondary	–
	EPSD [45]	68 organisms	p-site: ∼ 1,616,800 P: ∼ 209,300	Experimentally-validated; secondary	http://epsd.biocuckoo.cn
Specific database for phosphorylation	PhosphoNET [54]	Human	p-site: ∼ 966,000 P: ∼ 20,000	Experimentally-validated and predicted; secondary	http://www.phosphonet.ca
	RegPhos 2.0 [49]	Human, mouse, and rat	p-site: ∼ 113,000 P: ∼ 18,700	Experimentally-validated and predicted; secondary	http://140.138.144.141/∼RegPhos
	dbPSP [55]	200 prokaryotic organisms	p-site: ∼ 19,300 P: ∼ 8600	Experimentally-validated; secondary	http://dbpsp.biocuckoo.cn/
	pTestis [56]	Mouse	p-site: ∼ 17,800 P: ∼ 3900	Experimentally-validated and predicted; secondary	–
	LymPHOS [46]	Human and mouse	p-site: ∼ 18,300 P: ∼ 4900	Experimentally-validated and predicted; primary	http://www.lymphos.org
	P^3^DB [57]	45 plant organisms	p-site: ∼ 220,000 P: ∼ 57,000	Experimentally-validated and predicted; secondary	http://www.p3db.org

*Note*: This table was adapted from Table 1 [36] with permission. The type of database can be secondary or primary: secondary databases are the integration of other databases, and primary databases are independent. p-site, phosphorylation site; P, phosphoprotein; URL, uniform resource locator; PTM, post-translational modification; dbPTM, database of protein post-translational modifications; HPRD, Human Protein Reference Database; PHOSIDA, phosphorylation site database; PTM-SD, post-translational modification structural database; EPSD, eukaryotic phosphorylation site database; RegPhos, Regulatory Network in Protein Phosphorylation; P^3^DB, Plant Protein Phosphorylation Database.

### EPSD

**Figure 2:**
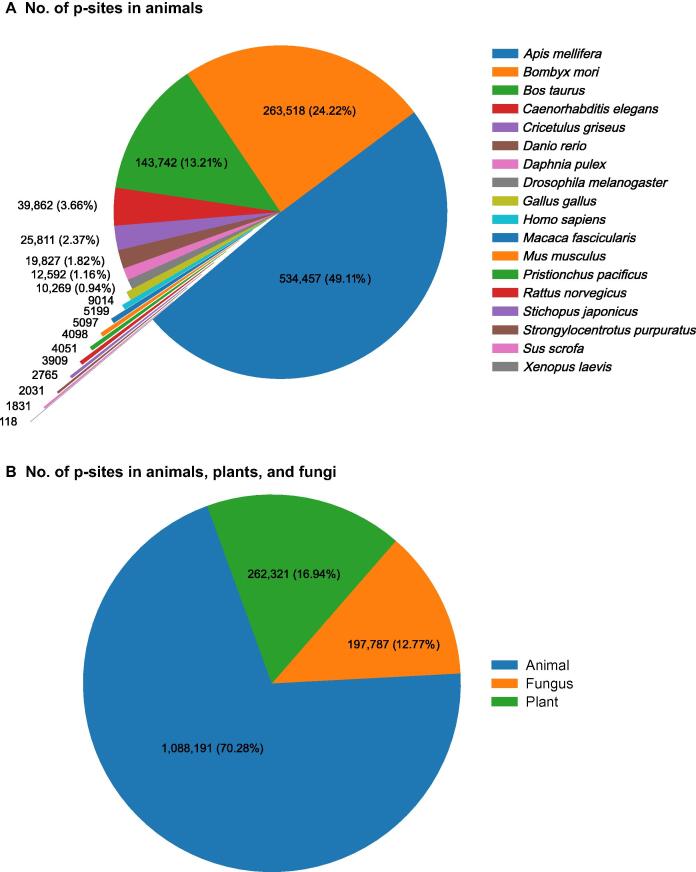
**EPSD database analysis** **A.** Number of p-sites in the animal proteins distributed by different animal species. **B.** Number of p-sites in proteins from animals, fungi, and plants. All figures are based on EPSD. p-site, phosphorylation site; EPSD, eukaryotic phosphorylation site database.

**Figure 3:**
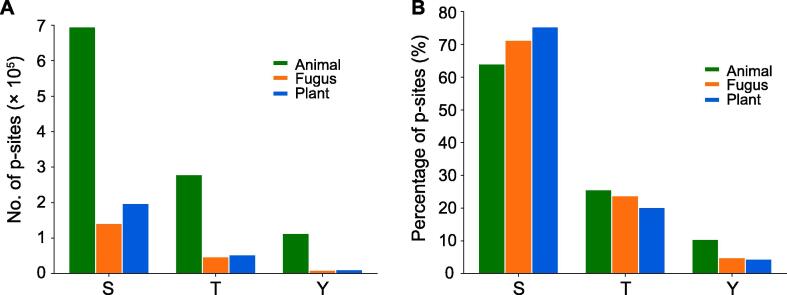
**Distribution of phosphorylated S, T, and Y inEPSD** Number (**A**) and percentage (**B**) of phosphorylated S, T, and Y in proteins from animals, plants, and fungi in EPSD. S, serine; T, threonine; Y, tyrosine.

**Figure 4:**
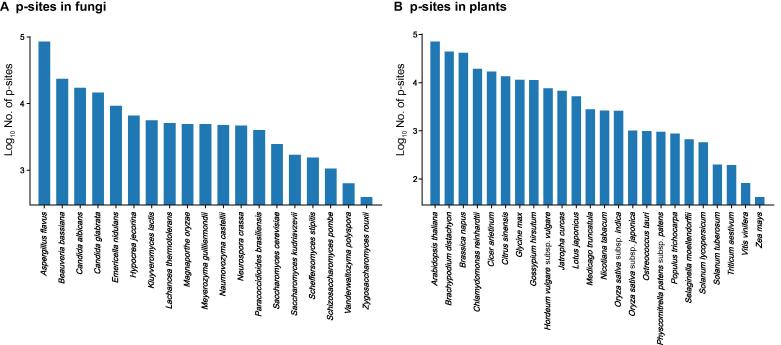
**p-site distribution in fungi and plants inEPSD** Number of p-sites in figui (**A**) and plants (**B**) in EPSD.

EPSD is one of the most specific and comprehensive databases for p-sites which has been updated in 2020. EPSD has been updated from two databases, the database of p-sites in plants (dbPPT) [58] and the database of p-sites in animals and fungi (dbPAF) [59], which includes roughly ∼ 82,000 p-sites from 20 plants and more than 483,000 p-sites from 7 different types of animals and fungi. Moreover, EPSD collects p-sites from 13 additional databases, including PhosphoSitePlus [50], Phospho.ELM [48], Universal Protein Resource (UniProt) [60], PhosphoPep [61], Biological General Repository for Interaction Datasets (BioGRID) [62], dbPTM, Fungi Phosphorylation Database (FPD) [63], HPRD, *Medicago* PhosphoProtein Database (MPPD) [64], Plant Protein Phosphorylation Database (P^3^DB) [57], phosphorylation site database (PHOSIDA) [53], *Arabidopsis* Protein Phosphorylation Site Database (PhosPhAt) [65], and SysPTM [38]. Totally, this database contains ∼ 1,616,800 experimentally known p-sites in ∼ 209,300 phosphoproteins of 68 eukaryotes (18 animals, 24 plants, 19 fungi, and 7 protists) [45]. Figure 2 shows the number of p-sites in different animal species (Figure 2A), and also depicts the distribution of p-sites in animals, fungi, and plants (Figure 2B) in the EPSD database. Figure 3 shows the number and the percentage of S, T, and Y p-sites in the EPSD database for animal, fungus, and plant species. Figure 4 shows the number of p-sites in different plant and fungus species in the EPSD database.

### dbPTM

A general database called dbPTM integrates PTM’s data from 30 databases and ∼ 92,600 research articles. dbPTM covers 130 types of PTMs in more than 1000 organisms [36]. The new version of dbPTM [66] in 2022 has curated more than 2,777,000 PTM sites from 41 published databases and ∼ 82,000 research articles.

### Identifying driver mutations and their effects on p-sites

Phosphorylation is involved in many aspects of cellular organization and signaling pathways associated with diseases. Various studies have demonstrated that p-sites are evolutionarily constrained in human genomes, as well as prevalent in cancer driver mutations and causal variants of inherited diseases. Therefore, phosphorylation information and knowledge of its function are useful for interpreting genetic variations, genotype–phenotype associations, and molecular diseases and their treatment [67].

The most common type of sequence change, DNA single nucleotide variants (SNVs), is caused by a single nucleotide change. Genetic variation of p-sites via SNVs can have an effect directly by modifying target residues or indirectly by modifying the consensus binding sequences (*i.e.*, short linear motifs) located in the flanking sequences of phosphorylated residues. As a result, this can change signaling networks by making, changing, and disrupting the p-sites [68]. There have been reports of phosphorylation-related SNVs that disrupt existing sites, create new sites, disturb kinase–substrate interactions, and cause disease phenotypes. A major challenge faced by biomedical research is the identification of genotype–phenotype associations, molecular mechanisms, and cancer driver mutations [67].

There are various databases with a useful list of genome variants in p-sites and other PTM sites. However, they provide no perspective on how mutations on p-sites and other protein sites will affect kinase binding [67], [68], [69]. Therefore, databases and updated tools are required to interpret rapidly increasing genomic and phosphoproteomic data to explain the signaling networks. We are briefly going to describe the ActiveDriverDB database as well as mutation impact on phosphorylation (MIMP) and PTMsnp tools in this field [67], [68], [69].

ActiveDriverDB is a web database that was designed to understand how protein coding varies in the human genomes. The ActiveDriverDB database contains more than 260,000 experimentally identified PTM sites in human proteome using public databases like PhosphoSitePlus, UniProt, Phospho.ELM, and HPRD, which contains ∼ 149,300 p-sites [42], [48], [50], [60]. As evidenced in the ActiveDriverDB database, changes in target amino acid substitutions in p-sites influence the creation of pathogenic disease mutations, somatic mutations in cancer genomes, and germline variants in humans. Additionally, the ActiveDriverDB database contains phosphoproteomics data reflecting the cellular response to severe acute respiratory syndrome coronavirus 2 (SARS-CoV-2) infection, which can be used to predict the impact of human genetic variation on SARS-CoV-2 infection and coronavirus disease 2019 (COVID-19) disease course [68].

An online tool called MIMP (http://mimp.baderlab.org/) can be used for predicting kinase–substrate interactions based on missense SNVs. MIMP analyzes kinase sequence specificities and predicts whether SNVs disrupt the existing p-sites or create new ones. This helps discover mutations that modify protein function by altering kinase networks and provides insights into disease biology and therapy development [67].

PTMsnp is another online tool for identifying driver genetic mutations aiming at PTM sites in proteins across different cohorts of TCGA by using a Bayesian hierarchical model. There are more than 411,500 modification sites in PTMsnp from 33 different types of PTMs and 1,776,800 mutation sites from 33 types of cancer. The web server detects proteins with a higher frequency of PTM-specific mutations in the motif region, considered to be the key targets in human disease development [69].

## Data gathering and preprocessing

In this section, we are going to describe steps concerning creating and preprocessing datasets before p-site prediction. In the last decade, due to the importance of phosphorylation in understanding the biological systems of proteins and in guiding basic biomedical drug design, research on phosphorylation has boomed. Several experimental methods are used to identify p-sites in a large number of phosphorylation examples with high accuracy (ACC), but many of them are labor-intensive and time-consuming. Therefore, low-cost and fast algorithmic and ML methods have become popular to overcome the problems associated with experimental methods [70]. In order to build a dataset for p-site prediction, all verified data from multiple databases are considered. Mainly, there are two main steps to prepare a dataset: data collection and data preprocessing [36], [70].

### Data collection

Data collection includes negative and positive data collection steps.

#### Negative data collection

S, T, and Y residues existing in experimentally-validated peptides without any phospho-groups are considered as non-p-sites or negative samples. There are two major strategies available to choose the negative samples. Firstly, from phosphoproteins, the negative random samples of the target residue that did not undergo the phosphorylation modifications are selected. Secondly, from non-phosphoproteins with none of their target residues (S, T, and Y) that have undergone specific phosphorylation (based on experimental evidence) are selected as the negative set [36], [71].

#### Positive data collection

S, T, and Y residues as p-sites or the positive samples are usually compiled from the aforementioned databases (*e.g.*, EPSD and dbPTM). These samples are usually known from experiments [36].

### Data preprocessing

After constructing the primary positive and negative datasets, one important task is removing inconsistent or redundant samples to gain a more reliable dataset.

Cluster database at high identity with tolerance (cd-hit) program is a protein clustering program widely used to reduce the sequence homology and filter out the similar ones. According to different phosphorylation prediction studies [70], [72], [73], [74], a threshold of identity is considered to range from 30% to 60% in many phosphorylation prediction studies [75].

There are three main steps in the literature for removing inconsistent or redundant proteins [36], [76]. First, redundant phosphoproteins should be removed by using the cd-hit program. In the second step, identical subsequences are removed within positive and negative sets by selecting the optimal window size. Finally, identical subsequences between the positive and negative datasets are removed by choosing the size of the optimal window size.

### Class-imbalance problem

**Figure 5:**
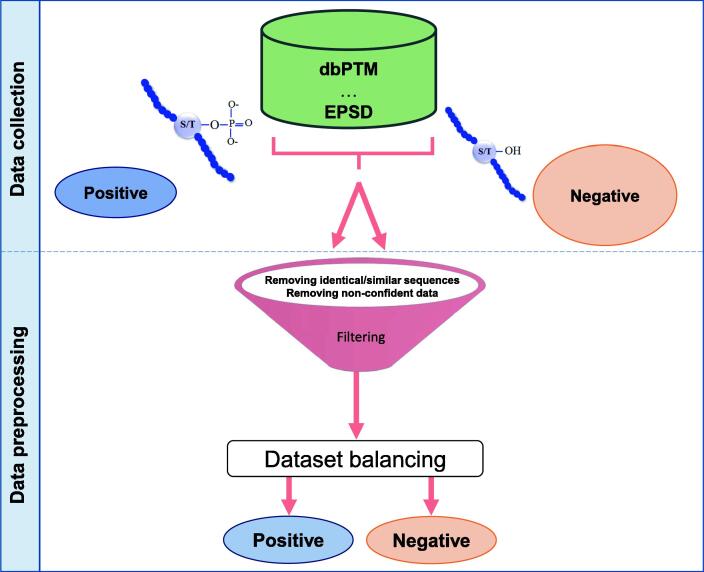
**Work flow of data collection andpreprocessing** Data preprocessing includes the balancing step. dbPTM, database of protein post-translational modifications.

It is a common problem in ML when there is an imbalance between the distribution ratios of data classes. In other words, a dataset that has unequal samples in classes is imbalanced. This is not an issue when the difference is not that much. Nevertheless, when one or more classes are infrequent, many models do not work well at identifying the minority classes. For example, in p-site prediction, preprocessed datasets are mostly imbalanced because the number of negative samples is much greater than the positive samples. Figure 5 shows the preprocessing framework for balancing data.

There are three most commonly used approaches to deal with class-imbalance problems: upsampling, downsampling, and customized loss function.

#### Upsampling

It generates additional data for minority classes either by making copies of the minimum class or by creating synthetic data which can represent samples of minimum classes.

#### Downsampling

It removes data from the majority class either randomly or using intelligent approaches of sample selection to handle the issue.

#### Customized loss function

This is a technique to deal with imbalance problems in ML that tries to customize the model loss function by assigning larger weights to minority. Customized losses have demonstrated better performance and attracted more attention than upsampling and downsampling approaches [77].

## Evaluation

The well-known evaluation metrics for protein p-sites are classified into five methods: ACC, sensitivity (SN), specificity (SP), Matthews coefficients of correlation (MCC), and the area under the receiver operating characteristic (ROC) curve (AUROC). These metrics are evaluated with a confusion matrix that summarizes the performance of models; it compares the real target values with those predicted by a model. The number of rows and columns in this matrix is based on the number of classes. From the confusion matrix, we will end up with four values [36], [76]. True positive (TP) represents the number of positive samples classified correctly. False positive (FP) represents the number of negative samples classified incorrectly. True negative (TN) represents the number of negative samples classified correctly. False negative (FN) represents the number of positive samples classified incorrectly.

### Model evaluation

**Figure 6:**
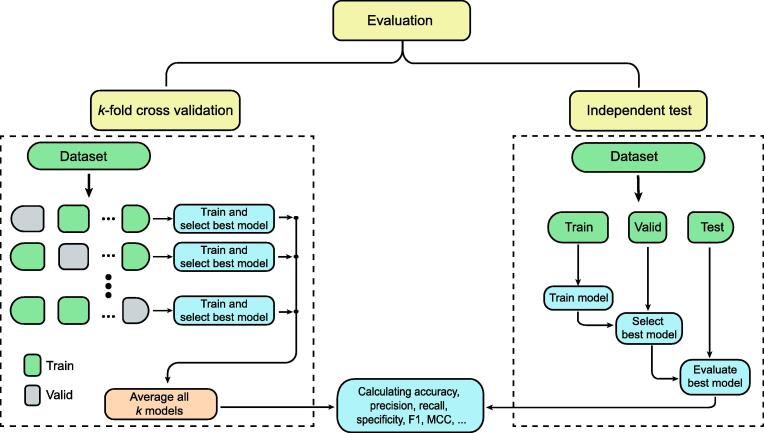
**Evaluation** The evaluation step can be done by two methods: *k*-fold cross validation and independent test. Independent test method sometimes is called “train–test” or “train–valid–test” as well. MCC, Matthews coefficients of correlation.

Basically, there are three methods for model evaluation for p-site prediction: independent test (train–test), *k*-fold cross validation, and jackknife cross validation (or leave-one-out cross validation). In the first one, a dataset is split into two sets: a train set and a test set. Then, the train set is divided into two subsets again: a train subset and a valid subset. The basic procedure is that the train subset is used to train models, and the valid subset is used for the evaluation of the trained models. After selecting the best model with respect to the valid subset result, we need to evaluate it on the test set. At the end, we should report the test set, and there shouldn’t be much difference between the valid subset and the test set results (Figure 6).

On the other hand, there is another assessment strategy utilized to assess ML models on restricted data samples. The method contains a single parameter called *k* that alludes to the number of bunches that data samples should be divided into. That is why the procedure is called *k*-fold cross validation, in which specific values for *k* can be chosen. Considering the scenario of 5-fold cross validation (*k* = 5), a dataset is divided into 5 bunches. Within the first iteration, the primary fold is utilized to assess the model, and the rest are utilized to train the model. Within the second iteration, the subsequent fold is utilized as the validation set, whereas the rest serve as the training set. This process is repeated until each fold has been used as the validation set. Each sample is given the opportunity to be utilized within the validation set one time as well as utilized to train the model *k*−1 times. The *k*-fold cross validation is usually used when the amount of train–valid data is limited. On the contrary, when dealing with huge amounts of data, we do not need to have a big valid set. In other words, the proportion of the train–valid split can sometimes go below 1% for the valid set. This approach is mostly used when massive amounts of data are accessible. However, in low-data regimes, they usually split with proportions of 30%–70%.

Note that there is also another evaluation strategy named the jackknife cross validation test [78], rarely used for p-site prediction. As the most objective method, the jackknife cross validation (or the leave-one-out cross validation) delivers unique results for a dataset in which one sample is selected to serve as the test data, whereas the rest are used as the training data. This procedure is repeated *N* times for a dataset with *N* samples, which can be expensive for large datasets [79].

In summary, *k*-fold should be used in low-data regimes, and an independent method with a small percentage of a test set should be used when we have access to lots of data.

## Methods for predicting p-sites

In the following sections, we are going to review methods of p-site prediction by dividing them into two main categories: algorithmic methods and ML. Likewise, ML methods are also divided into two approaches: conventional ML methods and end-to-end DL methods.

### Algorithmic methods

Innovative algorithms based on statistical approaches have been used in many studies. Here, we need to define algorithmic methods as computational methods in which there are no learning algorithms to gain information directly from data. Schwartz and Gygi [80] proposed a statistically repetitive method, using a set of phosphorylated peptide sequences to extract the patterns and a set of peptide sequences to evaluate the predictions. They mapped two sets of sequences to the position weight matrix so that in the matrices, the number of repetitions of each residue was determined from 6 positions higher to 6 positions lower than each p-site (which means their window size for each peptide is 13 amino acids long). Then, they formed a binary matrix based on these two matrices. This final matrix indicates the probability of observing a specific residue around a p-site by examining this matrix and comparing it with other p-sites.

Chen et al. [81] presented a new method for predicting p-sites by collecting four background datasets, including phosphorylated and non-phosphorylated sequences. They chose a given length of 13 amino acids for windows around p-sites. Initially, they formed the position weight matrices and then extracted the patterns. By scoring those patterns and deleting some of them, they finally reported a series of patterns as the output during an iterative cycle.

He et al. [82] showed that the number of patterns to be examined around each position is growing exponentially based on the length of the window. They referred to two developed algorithms to find phosphorylation patterns, named Motif-X and model-based DL (MoDL) algorithms. They supposed that these algorithms do not detect all patterns, and some patterns remain hidden from biologists. Therefore, they introduced a new algorithm called Motif-ALL to discover and report all possible patterns based on previous algorithms.

There has been a family of algorithms called Group-based Prediction System (GPS) for many years as algorithmic methods [83], [84], [85], [86], [87], [88], [89]. In 2004, an algorithm was developed for group-based p-site prediction and scoring 1.0, based on the hypothesis that similar short peptides exhibit similar biological functions. Likewise, the algorithm was refined and created an online service called GPS 1.1, which could predict p-sites for 71 PK clusters. Then, GPS 2.0 and 2.1 were presented with the same scoring strategy using two methods named matrix mutation (MaM) and motif length selection (MLS), which were designed to improve the ACC. Consequently, GPS 2.2, 3.0, 4.0, and 5.0 algorithms were developed, which are used for the prediction of other PTM sites rather than p-sites [31].

### ML methods

Most algorithms used for phosphorylation prediction are based on ML. Moreover, with explosions of the DL method in the early 2010s, ML has become even more popular than before. ML is generally the ability of machines to do actions based on prior knowledge and experience [90]. There are more than 40 different methods for predicting p-sites, and many of them are based on ML techniques, including logistic regression (LR), support vector machine (SVM), random forest (RF), and *k*-nearest neighbor (KNN) [70].

In general, there are two main strategies in ML to predict phosphorylation: conventional ML methods and end-to-end DL methods. The conventional approach stands for using ML algorithms as a part of solving a solution besides other steps in a pipeline design such as feature extraction and hand-feature engineering. In other words, usually, in a conventional ML-based system, there are multiple stages of processing which need to be designed individually. However, the end-to-end DL approaches can replace all those steps with a single neural network. This type of learning tries to eliminate the need for explicit feature engineering steps inside the learning system by feeding raw data as the input to it.

### Feature extraction

In protein phosphorylation prediction, various types of conventional approaches have been studied. Feature extraction is an important step in those approaches [91]. In this review, we summarized 20 feature extraction techniques suggested according to the physicochemical, sequence, evolutionary, and structural properties of amino acids. We have tried to introduce the most important and practical methods of feature extraction in the following.

#### Physicochemical property-based features

##### Encoding based on grouped weight

Encoding based on grouped weight (EBGW) divides 20 amino acids into 7 categories based on their hydrophobicity and charge characteristics [92], [93]. For each group *H_i_* (*i* = 1, 2, 3), a 25-dimensional array *S_i_* (*i* = 1, 2, 3) of the same element in the segment should be generated. If the amino acid at that position belongs to the *H*_*i*_ group, the element in the array will be set to 1; otherwise, it will be set to 0. Each array will be divided into sub-arrays (*J*-ones), which are represented as *D*(*j*). This value can be taken from cutting the main *S_i_* from the first window with *len*(*D*(*j*)) defined as Equation (1):

(1) $$len\left(D\left(j\right)\right)=int\left(\frac{j\times L}{J;}\right)\;\;\;j=1,2,\cdots ,J\;\;\;L=length\;of\;segments$$

For each group of *H_i_*, a vector with a length of *J* based on its sub-arrays should be defined, in which the *j*-th element of $X_{i}^{\left(j\right)}$, is calculated based on Equation (2):

(2) $$X_{i}^{\left(j\right)}=\frac{\mathrm{Sum}(D\left(j\right))}{\mathrm{len}(D\left(j\right))}\;$$

##### Amino acid index

The features based on amino acid index (AAINDEX) are extracted from the AAINDEX database. This database is used to represent various physicochemical and biochemical properties of each amino acid alone and also in pairs of them in every PTM [94]. The feature encodes 14 properties: hydrophobicity, polarity, polarizability, solvent/hydration potential, accessibility reduction ratio, net charge index of side chains, molecular weight, ionization equilibrium constant *pKa* (-COOH), ionization equilibrium constant *pKa* (-NH3), melting point, optical rotation, entropy of formation, heat capacity, and absolute entropy [92], [95].

##### Average accumulated hydrophobicity

Average accumulated hydrophobicity (ACH) quantifies the tendency of amino acids surrounding S, T, or Y residues to be exposed to solvent [96]. For different window sizes, ACH is calculated by averaging the cumulative hydrophobicity indices around the p-site. Note that every site is located in the center of the sliding windows [97], [98].

##### Encoding scheme based on attribute grouping

The encoding scheme based on attribute grouping (EBAG) represents the hydrophobicity attribute of the amino acids and divides the residues into 4 classes based on their physicochemical properties: hydrophobic class c_1_ = {A, F, G, I, L, M, P, V, W}, polar class c_2_ = {C, N, Q, S, T, Y}, acidic class c_3_ = {D, E}, and basic class c_4_ = {H, K, R} [99], [100].

##### Overlapping property

Overlapping property (OP) clusters each protein based on its chemical attributes. Each amino acid is classified into 10 physicochemical properties: polar, positive, negative, charged, hydrophobic, aliphatic, aromatic, small, tiny, and proline [98].

##### Pseudo amino acid composition

Pseudo amino acid composition (PseAAC) is first defined by Chou et al. [101] for coding proteins. They proposed sequence order and physicochemical information in protein sequences. For more details, refer to [102], [103], [104], [105].

#### Sequence-based features

##### Quasi-sequence order

Quasi-sequence order (QSO) describes the physicochemical distance between amino acids [92]. Most physicochemical properties are hydrophobicity, hydrophilicity, polarity, and side-chain volume. This feature was originally proposed by Chou et al. in [101]. For more detail, refer to [101], [106].

##### Numerical representation for amino acids

It converts each character of amino acids into numerical numbers by mapping them in alphabetic order from 1 to 20, and the dummy amino acid X represents 21 [92].

##### Binary encoding of amino acids

Binary encoding of amino acids (BINA) represents each amino acid as 21-dimensional binary vectors, which encodes 1 for the target amino acid and 0 for the residues (other 20 amino acids). For example, alanine (“A”) is shown as 100000000000000000000 [92].

##### Logo

This feature is defined by calculating the occurrence of amino acid frequencies and encoding them in a sequence with the Two Sample Logo program [92].

##### Position weight amino acid composition

Position information of each amino acid is another key point that shall be considered in feature extraction. Position weight amino acid composition (PWAA) can reveal sequence order information around P, S, and Y residues [107]. PWAA can be declared from Equation (3), in which *L* represents the number of upstream or downstream amino acids from p-sites in specific windows. If $x_{i,j}$ = 1, it means that each amino acid belongs to the *j*-th position in the window, otherwise $x_{i,j}$ = 0.

(3) $$C_{i}=\frac{1}{L\left(L+1\right)}\overset{L}{\underset{j=-L}{\sum }}x_{i,j}\left(j+\frac{\left|j\right|}{L}\right)\;j=-L,\cdots ,L\;$$

##### Composition of *k*-spaced amino acid pairs

The encoding of the composition of *k*-spaced amino acid pairs (CKSAAP) is pretty easy and can be directly calculated from the sequence pieces of p-sites and non-p-sites. CKSAAP is one of the important feature encoding schemes in lots of prediction tasks, especially in representing short sequence residues in protein sequences or subsequences. All 21 amino acids contain 441 different possible pairs. For scanning pieces to count all pairs of amino acids with *k*-space, we can use different window sizes. For example, window AXXV is a 2-space amino acid pair in *k* = 2 [73], [108]. The CKSAAP equation is proposed as Equation (4) [73]. In this equation, *L* denotes the length of the window, and $A_{i}A_{j}$ is an amino acid pair.

(4) $$f_{i,j}=\frac{Num\left(A_{i}A_{j}\right)}{L-K-1}\;i,j=1,2,\cdots ,21$$

##### Amino acid composition

Amino acid composition (AAC) is the most commonly used feature, which simply calculates the frequency of each amino acid in subsequences of a protein while encoding the information into 20 bits [109]. This feature is also represented as amino acid frequency (AF) in some research. Both AF and AAC reflect the frequency of each amino acid or amino acid pair’s occurrence. Lin et al. [109] proposed the AAC equation as Equation (5), in which $c_{i}$ is the number of amino acid *i* in the sequence and $v_{i}$ refers to AAC.

(5) $$v_{i}=\frac{c_{i}}{len\left(seq\right)}\;i=1,\cdots ,20$$

#### Evolutionary-based features

##### KNN

The most popular feature selection method that is used in various ML problems, especially in PTM and phosphorylation classification, is KNN. It classifies sequences based on their distance. The algorithm classifies sequences by looking at *k* of nearest neighbor sequences and finding out the majority of votes from nearest neighbors that have similar attributes and the shortest distance as those used to map the items [110].

##### Position-specific scoring matrix-based transformation

Position-specific scoring matrix-based transformation (PSSM) encodes the evolutionary data of a protein, which is very informative and useful for some biological classification problems. The PSSM matrix in a protein with a sequence of length *L* is a matrix with *L* × 20 dimensions. In the matrix, each row represents an amino acid in the protein sequence, and the columns represent the 20 amino acids in proteins [111].

#### Structural-based features

##### Protein disorder features

All PTMs include p-sites located within disorder positions [112]. Protein disorder features (DFs) were used as features in many studies [97], [113], [114].

##### Shannon entropy

Shannon entropy (H) in information theory quantifies the amount of uncertainty of a random variable. To be more precise, it is the average (expected value) amount of information obtained from observing a random variable. It means that when the entropy of a random variable is high, we have more ambiguity about that random variable [115]. In science and engineering in general, entropy is a measure of the degree of ambiguity or disorder [116].

##### Relative entropy

Relative entropy (RE) is known as Kullback–Leibler which is aggregated entropy for more than 20 sites in proteins [74].

##### Information gain

Information gain (IG) can be computed by subtracting RE from H [Equation (6)] [98].

(6) IG=H-RE

##### Accessible surface area

Accessible surface area (ASA) or solvent-ASA is a biomolecule surface that can access the solvent. This is an essential structural feature determining the protein’s folding and stability [98].

### Conventional ML approach

Once the features have been extracted, classification models should be adopted to predict the p-sites. One of the most popular classifiers is SVM [97], [109], [117].

SVM is a linear model for classification and regression problems that uses a line or hyperplane to separate data. In other words, SVMs calculate the maximum margin boundary that leads to the equivalent division of all data points. First, SVM uses a line to classify each data point based on their distance. If data points are not linearly separable in low-dimensional space, there may be multiple transformations enabling the data to be linearly separable in higher dimensions. Therefore, SVMs can find a hyperplane in higher dimensions between different classes of data such that the distance between data points falling on either side of that hyperplane is maximized [118], [119]. Nowadays, SVMs have been widely used in bioinformatics, especially in PTM problems [97], [107], [120].

**Figure 7:**
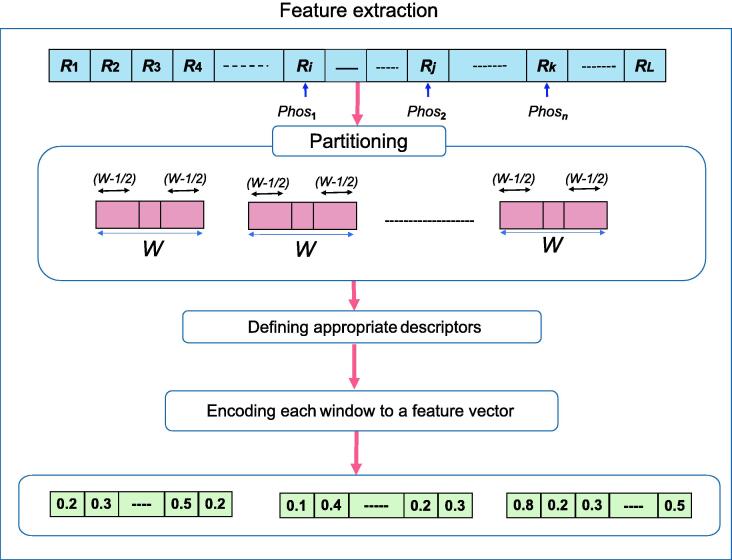
**A common procedure of featureextraction**

**Figure 8:**
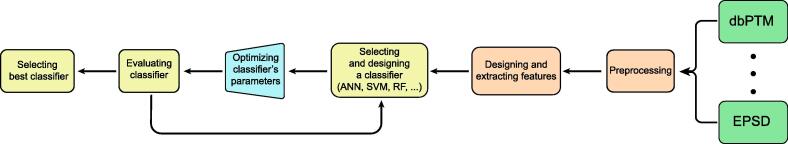
**Conventional MLprocedure** ML, machine learning; ANN, artificial neural network; SVM, support vector machine; RF, random forest.

RF is another well-known and important classifier frequently used in this field. This algorithm can randomly build a forest that contains a large number of decision trees. Each tree constructs a class prediction, and the class with the most votes will become the model prediction [121]. Figure 7 demonstrates the procedure of feature extraction, and Figure 8 shows the process of conventional ML methods.

As of recent years, kinase-specific methods have been used since, in general, some protein prediction sites have not yet been explored and kinases can assist in locating these sites. NetPhos [122] and NetPhosK [123] both used deep neural networks (DNNs) based on consensus sequences and MS experimental methods. These algorithms are specific to the kinase’s family. In the Quokka framework [30], the LR approach was suggested to classify 43 S/T and 22 Y kinase family sites. Kim et al. [117] proposed to use the consensus sequence structure as features and a SVM classifier to predict four kinase groups and families. The best ACCs achieved by their model were reported around 83%–95% at the kinase family level and 76%–91% at the kinase group level. Liu et al. [124] proposed a method for prediction of four kinase families based on RF, which extracted features with an auto covariance (AC) transform and seven physicochemical properties and achieved over 90% ACC.

To recognize protein p-sites in universal proteins, Huang et al. [107] proposed a method based on SVM in viruses. They used EBAG and PWAA features for extracting the physicochemical and sequence information of viral proteins around p-sites. They used 10-fold cross validation and an independent test set for different window sizes ranging from 15 to 27 amino acids. They got the best results for window size of 23 amino acids with ACC scores of 88.8%, 95.2%, and 97.1% for S, T, and Y sites, respectively. They also showed the influence of using different features. Their model improved almost 15% when they used the combination of two EBAG and PWAA features.

Furthermore, Lin et al. [109] used KNN, AF, and CKSAAP as features and combined different features together to feed it into their model to investigate the best features. The combination of AF and CKSAAP provided the best ACC for their SVM model. They believed SVM could classify rice proteins as universal p-sites. Their work was named Rice_Phospho 1.0, which achieved 82% ACC.

Cheng et al. [97] proposed a granular SVM (GSVM) for predicting universal p-sites. They used KNN, AF, and DF features in every p-site position to make the train set. To split data into high-dimensional feature spaces, they used kernel fuzzy C-means clustering as a feature extraction method. The method was applied to plant and animal dataset types and could achieve 80% and 85% ACC scores, respectively.

By using the PhosPred-RF method, Banerjee et al. [71] used information extracted from PSSM and trained individuals with RF with odd window sizes ranging from 9 to 25 amino acids. They got approximately 70% ACC for 26 protein sequences. RF-Phos 1.0 transformed each amino acid into vectors by using eight algorithms of feature selection (H, RE, ASA, OP, ACC, QSO, and the sequence order coupling number of each sequence) based on a window size of 9 amino acids. They specifically showed which features are the most important and have more effects on ACC. It was mentioned that AAC was the best feature for S and T sites. Then, these features were used as RF input with 10-fold cross validation. The ACC of the model was approximately 80% for S, T, and Y sites [74]. Moreover, in the RF-Phos 2.0, their RF model was improved by using window sizes of 5 to 21 amino acids and using different features. QSO was the best feature for S and T sites [98]. RF-Phos 1.0 and RF-Phos 2.0 specifically predicted universal p-sites. It should be mentioned that feature selection methods helped to improve the ACC of various approaches.

Microbial Phosphorylation Site predictor (MPsites) was proposed by Hasan et al. [125] to recognize universal microbial p-sites with different sequence features. In order to convert each sequence to numerical vectors, they used various sequence encoding strategies, including AF, BINA, AAINDEX, and PWAA. They used naïve bayes, SVM, neural networks, decision trees, and RF algorithms to recognize S and T p-sites. Results showed that RF has better performance than the other algorithms. It got 68% ACC for S sites and 75% ACC for T sites [125].

Cao et al. [126] proposed a method named PreSSFP to predict p-sites in seven species-specific fungi proteins. They used a strategy including two steps for feature optimization to improve the SVM prediction performance. KNN, AAC, di-amino acid composition, and physiochemical properties were used as features. First, with the RF model, they sorted each input feature based on the mean ACC. In the second step, the top ten features from the previous step were merged to train the SVM model. Finally, they achieved over 80% ACC.

Chen et al. [127] proposed a feature selection method named ga-aided ant colony system (GAS), based on ant colony and genetic algorithms, for the classification of six kinase types.

Qiu et al. [105] developed an approach called iPhos-PseEvo. Protein sequence evolutionary and PseAAC were selected as features for an ensemble RF model. The ACC for their model was 71% with the jackknife test evaluation approach.

As a final example in this section, multi-iPPseEvo [104] is similar to iPhos-PseEvo but with a different implementation strategy while using *k*-fold cross validation. This method contains a multi-ensemble RF classifier for each S, T, and Y site and proposes multi-label p-site prediction for each site.

### End-to-end DL approach

**Figure 9:**
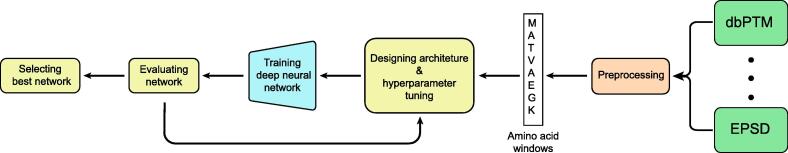
**End-to-end DLprocedure** DL, deep learning.

End-to-end learning has become a hot topic in the ML field by taking advantage of DL. DNN is almost the same as traditional artificial neural networks (ANNs), which is composed of many connected neurons that work together to solve specific issues, inspired by the functionality of biological neural networks in the human brain. Inspired by the human brain, each DNN’s layer (or group of layers) could be used for learning the hierarchical abstraction for downstream tasks. In other words, usually raw input sequences are just fed to a DNN, and the process of feature selection automatically happens between layers. Since it refers to training a possibly complex learning system by applying gradient-based learning to the system as a whole, it is called end-to-end DL. These systems are specially designed so that all components are created to be differentiable, and consequently, learnable. That is to say, it is a procedure in which a model learns all the steps, including feature selection and extraction, between the first and last layers [128]. Figure 9 shows the common procedure of end-to-end DL methods.

Need to mention that in order to prepare a sequence of amino acids for the end-to-end DL system, there are two prerequisite steps [129]: (1) sequence encoding, and (2) converting the encoded sequence to numerical vectors. The second step could be done via either one-hot encoding [72] or another popular technique named word embedding [130]. Therefore, one-hot encoding is not considered a feature extraction method and is simply used to represent categorical inputs (*e.g.*, amino acid codes) into numerical vectors in order to feed to DL models. However, in an end-to-end DL network, word embedding is often used in PTM due to the similarity between PTM and natural language processing (NLP) domains as well as the effectiveness of the technique.

We have shown a great success of DL in solving problems in different domains of science, especially in biological problems with finding non-obvious patterns or making predictions in datasets [130], [131], [132], [133], [134], [135], [136]. In recent years, DL has been applied to PTM classification of proteins, such as p-site prediction. As mentioned earlier, the main aspect of this approach compared with the conventional ML approach is that the feature extraction step is not designed by human engineers or manually. These layers are acquired from input data to extract the best patterns accurately and quickly. Though the most important point about DL is that it needs huge amounts of data, by increasing the size of the dataset, it can perform better. This can be counted as a drawback; when the dataset is not big enough, it quickly falls behind other ML methods in terms of performance.

Among all DL architectures, convolutional neural networks (CNNs), recurrent neural networks (RNNs), and long short-term memory (LSTM) are the most famous [70], [72], [137].

Wang et al. [72] provided a DL architecture called MusiteDeep to predict general and kinase-specific families’ positions in a sequence. The window size used for the input sequence was 33 amino acids. Then, they presented their network with a multi-layer CNN and attention layer architecture. In contrast to multi-layer models of MusiteDeep, DeepPhos [70] used dense CNN blocks that could show different and multiple representations of proteins for p-site predictions by using the concatenation of intra-block layers and inter-block layers. The method could improve the performance of MusiteDeep by using different window sizes with lengths of 15, 33, and 51 amino acids. Both of these methods, DeepPhos and MusiteDeep, have been developed for kinase families and universal p-sites. Moreover, PhosTransfer [137] is a DL-based framework that constructs a pre-train architecture with CNNs based on hierarchy kinases systems and transfer learning. It was specialized for improving kinase p-site prediction. The method was to accumulate the information of a hierarchical kinase’s classification tree at family, subfamily, and group levels. It could achieve AUROC of 0.89 on average. The DeepPPSite is another DL model based on universal p-site prediction with consideration of sequence information [73]. Ahmed et al. used one-hot encoding sequence as input, PSSM, EBGW, CKSAAP, and AAINDEX as features, and stacked LSTM architecture as a predictive model. The MCC values reported for S, T, and Y are 0.358, 0.356, and 0.350, respectively.

Awais et al. [32] developed a computational model named iPhosH-PseAAC using an ANN algorithm to predict PhosH sites in protein sequences. The model was based on features such as PseAAC, statistical moments, and position-relative features. To validate the iPhosH-PseAAC predictor, they performed self-consistency testing, 10-fold cross validation, and jackknife test, which resulted in ACC scores of 100%, 94.26%, and 97.07%, respectively. Wang et al. [93] proposed a hybrid model named MaloPred for predicting PhosH sites in the proteome. The model was composed of two CNN-based classifiers and a RF-based classifier and was trained on three types of features: one-of-K coding, enhanced grouped amino acid content (EGAAC), and composition of *k*-spaced amino acid group pairs (CKSAAGP) encoding. They found that MaloPred was able to accurately predict PhosH sites from sequence information through both 10-fold cross validation and independent tests.

Furthermore, there have been some researches such as the work of Lv et al. [138] that used hybrid architectures. They presented a specific hybrid end-to-end architecture that combined both CNN and LSTM together, called DeepIPs, to predict universal p-sites in host cells infected with SARS-CoV-2 [139], [140]. Lv et al. utilized three approaches in NLP as word embedding layers to represent amino acids as vectors: GloVe [141], FastText [142], [143], and Word2vec [129] pre-training word embedding methods. The final ACC for this method was reported as 80.45% for S/T and 75.22% for Y.

DL provides a highly effective framework for dealing with modern-day learning challenges. The modern high-performance interpretable deep tabular learning network (TabNet) provides an extremely powerful framework for solving more challenging learning problems [144]. For example, Khalili et al. [76] developed a TabNet model to predict p-sites in soybean with a high ACC rate that outperformed other common ML methods (LR-L1, LR-L2, RF, SVM, and XGBoost). They assessed and compared the strength and reliability of all models using 10-fold cross validation. Experiments assessed the performance of AAC, dipeptide composition (DPC), tripeptide composition (TPC), PSSM, and physicochemical properties as individual features. To extract training sequences for model development, various window sizes ranging from 7 to 35 amino acids were used. They got the best results for window size of 13 amino acids with an ACC of 87.34% based on PSSM features.

Naseer et al. [145] compared human-based feature representation with DL-based representation for the reorganization of phosphoserine p-sites. The combination of the RNN–LSTM model got 81.1% ACC, and the CNN-based model achieved 78.3% ACC. In contrast to human engineering with 77% ACC, DL methods have performed better for phosphoserine p-site prediction.

Even though most DL approaches worked well with large volumes of data, a study [26] with a small amount of data from only two kinase families proposed a simple DNN architecture and achieved around 80% ACC. It means that end-to-end learning can also perform successfully in low-data regions. This algorithm was designed for both the kinase families and universal p-sites.

Guo et al. [146] collected phosphoprotein-binding domains (PPBDs) that interact with PPBD-containing proteins (PPCPs) from 12 eukaryotic species and developed a DNN framework based on transfer learning to classify the protein binding domains into a hierarchical structure with three levels, including group, family, and single PPBD cluster.

Despite most end-to-end approaches using raw sequences (one-hot encoding) as input, PhosIDN [147] trained a DNN by combining raw sequences and PPI information together. This architecture contains three sub-networks: (1) sequence feature encoding sub-network (SFENet), (2) PPI feature encoding sub-network (IFENet), and (3) heterogeneous feature combination sub-network (HFCNet).

### p-site prediction tools

Due to the high cost and low speed of using experimental methods to recognize p-sites, in recent years, many computational online tools have been developed to help increase the quality of p-site prediction. Table 2 introduces famous publicly accessible online tools or GitHub repositories for p-site prediction.

**Table 2:** Summary of p-site prediction tools

Tool	**Type /description**	**Method**	**Feature extraction method**	**Dataset size**	**Window size(amino acid)**	**Negative dataset**	**Unbalance strategy**	**Redundancy threshold**	**Evaluation strategy**	**URL**	**U/K**
NetPhos [122]	Conventional	ANN	Sequence composition features	902 p-sites	21 (Y, S) 25 (T)	–	–	–	5-fold	http://www.cbs.dtu.dk/services/NetPhos/	K
Kim et al. [117]	Conventional	SVM	–	855 p-sites on S, 216 p-sites on T	3–25	Phosphoproteins	Downsampling	70%	7-fold	http://www.ngri.re.kr/proteo/PredPhospho.html	K
Liu et al. [124]	Conventional	RF	Auto covariance transform, 7 physicochemical properties	1911 p-sites	–	Phosphoproteins	Downsampling	40%	5-fold, independent test	–	K
Huang et al. [107]	Conventional	SVM	EBAG, PWAA	230 p-sites on S, 61 p-sites on T, 14 p-sites on Y	23	Phosphoproteins	Downsampling	–	10-fold, independent test	–	U
Rice_Phospho 1.0 [109]	Conventional	RF	AF, CKSAAP, KNN	4220 p-sites on S, 605 p-sites on T, 141 p-sites on Y	25	Phosphoproteins	Downsampling	–	10-fold, independent test	http://bioinformatics.fafu.edu.cn/rice_phospho1.0	U
GSVM [97]	Conventional	SVM	KNN, AF, DF	∼ 50,000 p-sites	13	Phosphoproteins	Downsampling	30%	10-fold	–	U
RF-Phos 1.0 [74]	Conventional	RF	H, RE, ASA, OP, AAC, QSO	∼ 28,000 p-sites	5–21	Phosphoproteins	Downsampling	30%	10-fold, independent test	–	U
RF-Phos 2.0 [98]	Conventional	RF	H, RE, IG, ASA, OP, AAC, QSO	∼ 28,000 p-sites	5–21	Phosphoproteins	Downsampling	30%	10-fold, independent test	http://bcb.ncat.edu/RF Phos/	U
PhosTransfer [137]	Conventional	CNN	H, RE, DF, OP	∼ 10,000 p-sites on S, ∼ 34,000 p-sites on T, ∼ 3000 p-sites on Y	–	–	Downsampling	40%	Independent test	https://github.com/yxu132/PhosTransfer	K
DeepPPsite [73]	Conventional	LSTM	CKSAAP, EBGW, PSSM	∼ 7000 p-sites on S, ∼ 2000 p-sites on T, ∼ 700 p-sites on Y	15, 19, 21	Phosphoproteins	Downsampling	30%	10-fold, independent test	https://github.com/saeed344/DeepPPSite	U
GPS 5.0 [31]	Conventional	LR	Structural features	∼ 15,000 p-sites	20	Phosphoproteins	Downsampling	–	10-fold	http://gps.biocuckoo.cn	K
MPSite [125]	Conventional	RF	AF, OP, PSSM, PWAA	∼ 2700 p-sites on S, ∼ 2100 p-sites on T	7–25	Phosphoproteins	Downsampling	30%	10-fold, independent test	http://kurata14.bio.kyutech.ac.jp/MPSite/	U
Quokka [30]	Conventional	LR	KNN, AF, BLOUSM64	∼ 2400 p-sites on S, ∼ 370 p-sites on T	15, 19, 21	Phosphoproteins	Downsampling	30%	5-fold, independent test	http://quokka.erc.monash.edu/#webserver	K
PhosContext2vec [148]	Conventional	SVM	H, BLOUSM64, DF, OP, ACH, secondary structure	Universal: ∼ 20,000 p-sites on S, ∼ 5600 p-sites on T, ∼ 2100 p-sites on Y Kinases: ∼ 4100	25	Phosphoproteins	Downsampling	–	10-fold, independent test	http://phoscontext2vec.erc.monash.edu/	K/U
PhosphoSVM [120]	Conventionlal	SVM	H, RE, secondary structure, DF, ASA, OP, KNN	∼ 25,000 p-sites on S, ∼ 7200 p-sites on T, ∼ 2700 p-sites on Y	15, 19, 21	Phosphoproteins	Downsampling	30%	10-fold, independent test	http://sysbio.unl.edu/PhosphoSVM/	U
PhosPredRF [71]	Conventional	RF	H, RE, OP	∼ 4300 p-sites on S, ∼ 2700 p-sites on T	15, 19, 21	Phosphoproteins	Downsampling	30%	10-fold, independent test	http://bioinformatics.ustc.edu.cn/phos_pred/	U
PreSSFP [126]	Conventional	SVM	Sequence information, evolutionary information, physicochemical properties	Various for organisms	–	Phosphoproteins	Downsampling	30%	10-fold, independent test	http://computbiol.ncu.edu.cn/PreSSFP	U
GasPhos [127]	Conventional	Multiple classifiers	GAS	∼ 3400 p-sites	–	Phosphoproteins	Downsampling	–	5-fold	http://predictor.nchu.edu.tw/GasPhos	K
iPhos-PseEvo [105]	Conventional	Ensemble RF	KNN, PseAAC	845 p-sites on S, 386 p-sites on T, 249 p-sites on Y	–	Phosphoproteins	Downsampling	50%	Jackknife test	http://www.jci-bioinfo.cn/iPhos-PseEvo	U
Multi-iPPseEvo [104]	Conventional	RF	KNN, PseAAC	845 p-sites on S, 386 p-sites on T, 249 p-sites on Y	–	Phosphoproteins	Downsampling	50%	5-fold	http://www.jci-bioinfo.cn/Multi-iPPseEvo	U
DeepIPs [138]	End-to-end	CNN-LSTM	–	5387 p-sites on S/T, 102 p-sites on Y	33	Phosphoproteins	Downsampling	30%	Independent test	https://github.com/linDinggroup/DeepIPs. http://lin-group.cn/server/DeepIPs/	U
DeepPhos [70]	End-to-end	CNN	–	140,000 p-sites on S/T, 27,000 p-sites on Y	15, 33, 51	Phosphoproteins	Downsampling	40%	10-fold, independent test	https://github.com/USTCHIlab/DeepPhos	U/K
MusiteDeep [72]	End-to-end	CNN + attention	–	∼ 35,000 p-sites on S/T, ∼ 2000 p-sites on Y	33	Phosphoproteins	Downsampling	50%	5-fold, independent test	https://www.musite.net/ https://github.com/duolinwang/MusiteDeep_web	U/K
Lumbanraja et al. [26]	End-to-end	DNN	–	∼ 1800 p-sites on S, ∼ 700 p-sites on T, ∼ 200 p-sites on Y	9	Phosphoproteins	Downsampling	20%	10-fold	–	U/K
PhosIDN [147]	End-to-end	SFENet + IFENet + HFCNet	PPI graph embedding	∼ 160,000 p-sites	15, 33, 71	Phosphoproteins	Downsampling	40%	Independent test	https://github.com/ustchangyuanyang/PhosIDN	U/K
Khalili et al. [76]	Neural network + feature	TabNet	AAC, DPC, PSSM, physicochemical properties	∼ 4500 p-sites	7–35	Phosphoproteins	Downsampling	40%	10-fold	https://github.com/Elham-khalili/Soybean-P-sites-Prediction	U
iPhosH-PseAAC [32]	Neural network + feature	ANN	PseAAC	∼ 1300 histidine p-sites	–	Phosphoproteins	Downsampling	–	10-fold, jackknife test	–	U/K
PROSPECT [33]	End-to-end + Conventional	CNN + RF	One-hot encoding, EGAAC, CKSAAGP	∼ 1600 histidine p-sites	27	–	–	40%	10-fold, independent test	http://PROSPECT.erc.monash.edu/	U/K

*Note*: U stands for universal which includes all types of p-sties; K stands for kinase which includes only kinase-specific p-sites. ANN, artificial neural network; LR, logistic regression; SVM, support vector machine; RF, random forest; KNN, k-nearest neighbor; CNN, convolutional neural network; LSTM, long short-term memory; EBAG, encoding scheme based on attribute grouping; PWAA, position weight amino acid composition; AF, amino acid frequency; CKSAAP, composition of *k*-spaced amino acid pairs; DF, protein disorder feature; H, Shannon entropy; RE, relative entropy; ASA, solvent accessible surface; OP, overlapping properties; AAC, amino acid composition; QSO, quasi-sequence order; PseAAC, pseudo amino acid composition; PPI, protein–protein interaction; –, not available.

## Current limitations

In general, it is unfair to compare different ML algorithms applied to p-site prediction tasks to choose the best technique due to variation in preprocessing steps, evaluation methods, and, more importantly, database diversity in the literature. Therefore, we tried to evaluate several tools by creating three new test datasets. For this purpose, we selected the newly released version of the dbPTM [66] database in 2022 and picked up all new phosphoproteins in all organisms that did not exist in the previous versions. Subsequently, we built 161-all, 161-humans, and 100-top test sets.

In the 161-all set, 161 new proteins with p-sites were randomly selected from 161 newly released organisms’ proteins (one protein per organism). This test set consists of 13,403 sites, 402 of which are p-sites. The maximum and minimum length of sequences are 7096 and 49 amino acids, respectively.

In the 161-humans set, 161 proteins with p-sites were randomly selected from newly released proteins of *Homo sapiens*. This test set consists of 7383 sites, 714 of which are p-sites. The maximum and minimum length of sequences are 921 and 714 amino acids, respectively.

In the 100-top set, 100 new proteins with p-sites were randomly selected from the top 10 organisms that had the biggest new protein numbers (ten proteins per organism). This test set consists of 9321 sites, 507 of which are p-sites. The maximum and minimum length of sequences are 3498 and 102 amino acids, respectively.

Next, we tried to evaluate several universal p-site prediction tools introduced in Table 2 on these datasets. However, there were many hurdles in the evaluation stage. Kim et al. [117], RF-Phos 2.0, PhosPred-RF, PreSSFP [126], iPhos-PseEvo, and Multi-iPPseEvo were not available. Moreover, Rice_Phospho 1.0 and PhosphoSVM only take one sequence as input in order to process, and since the process was time-consuming, we could not evaluate our three test datasets on them. Furthermore, DeepIPs did not have any response to our request. Finally, we selected three tools — MusiteDeep [72], PhosIDN [147], and NetPhos [122] — to evaluate. By the way, since NetPhos [122] could not predict sequences with a length more than 4000 amino acids, and the 161-all test set had proteins more than that length, we could not evaluate the performance of NetPhos on the 161-all test set. Table 3 shows the results.

**Table 3:** Online tool evaluation

**Test set**	**MusiteDeep**[72]			**PhosIDN**[147]			**NetPhos**[122]		
	**161–all**	**161–humans**	**100–top**	**161–all**	**161–humans**	**100–top**	**161–all**	**161–humans**	**100–top**
TP	168	194	150	249	308	297	–	447	339
FP	1656	745	1044	4356	1140	2597	–	3701	5349
TN	11,378	5927	7781	8678	5532	6228	–	2971	3476
FN	201	517	346	120	403	199	–	264	157
ACC (%)	86.14	82.91	85.09	66.60	79.10	70.00	–	46.30	40.93
Precision	0.09	0.21	0.13	0.05	0.21	0.10	–	0.11	0.06
Recall	0.46	0.27	0.30	0.67	0.43	0.60	–	0.63	0.68
F1	0.15	0.24	0.18	0.10	0.29	0.18	–	0.18	0.11
Specificity	0.87	0.89	0.88	0.67	0.83	0.71	–	0.45	0.39

*Note*: TP, true positive; FP, false positive; TN, true negative; FN, false negative; ACC, accuracy; –, not available.

As shown in Table 3, all three tools performed weakly compared with the performances reported in their related studies [72], [122], [147]. We interpreted from the results that there are no valid benchmarks for p-site prediction. In other words, each study proposed a method applied to a unique test set to report the results, which makes it difficult to compare different methods together. Therefore, for fair and precise competition, we suggest that uniform, comprehensive, unique, and well-defined test benchmarks for p-site prediction will be prepared as a crucial step for future research in this field.

## Conclusion

Almost all proteins contain phosphorylation, which is responsible for critical functions in the cell. Various diseases can be caused by disruptions of this modification. The discovery of phosphorylation as one of the most important PTMs by high-throughput experimental methods is labor-intensive and time-consuming. Therefore, it is urgent to develop a tool or method to automatically predict the p-sites. As we investigated the literature, there is not a complete review for p-site predictions based on ML algorithms. Due to the importance of the issue, this review briefly introduces some popular PTM databases (including phosphorylation), methods, and online tools for p-site prediction to provide a guide to current research.

In this review, we introduced two important databases: EPSD and dbPTM, while comparing them in terms of p-site distribution. Then, we gave a brief overview of protein p-site prediction by ML techniques, which are mainly divided into classical ML and end-to-end DL methods. In addition to ML, we slightly discussed algorithmic methods as well. Algorithmic methods have a statistical basis which are slow and have high time complexity. On the other hand, ML algorithms, which are quite popular these days, have attracted a lot of attention in p-site prediction including SVM, LR, and RF. In conventional methods, SVM has shown better performance, although the feature extraction step would obviously have a significant impact on the final result. Therefore, this study introduced 20 important and widely used feature extraction methods based on structural, sequence, evolutionary, and physicochemical properties. Additionally, CNN- and RNN-based architectures, known as efficient end-to-end learning styles, were introduced, which are able to predict p-sites directly from the raw input sequences without any feature extraction steps.

In the next stage, the evaluation methods for predicting p-site approaches were reported to give the standard metrics for comparison between performances. Finally, in order to demonstrate the current limitations of p-site prediction methods, we created three test sets and evaluated several available online tools. All those methods performed poorly compared with the performances reported in their related studies [72], [122], [147], suggesting the importance of creating uniform and well-defined benchmarks for p-site prediction.

## Competing interests

The authors have declared no competing interests.

## CRediT authorship contribution statement

**Farzaneh Esmaili:** Conceptualization, Investigation, Data curation, Visualization, Writing – original draft. **Mahdi Pourmirzaei:** Investigation, Data curation, Formal analysis, Visualization, Software, Writing – review & editing. **Shahin Ramazi:** Investigation, Visualization, Writing – review & editing, Project administration. **Seyedehsamaneh Shojaeilangari:** Writing – review & editing. **Elham Yavari:** Writing – review & editing. All authors have read and approved the final manuscript.
